# Local recurrence in malignant peripheral nerve sheath tumours: multicentre cohort study

**DOI:** 10.1093/bjsopen/zrae024

**Published:** 2024-04-15

**Authors:** Christianne Y M N Jansma, Ibtissam Acem, Dirk J Grünhagen, Cornelis Verhoef, Enrico Martin, MONACO Collaborators, J Henk Coert, Uta E Flucke, Willem-Bart M Slooff, Thijs van Dalen, Lukas B Been, Han J Bonenkamp, Monique H M E Anten, Martinus P G Broen, Marc H A Bemelmans, Jos A M Bramer, Gerard R Schaap, Arthur J Kievit, Winan J van Houdt, Jos van der Hage, Michiel A J van de Sande

**Affiliations:** Department of Surgical Oncology and Gastrointestinal Surgery, Erasmus MC Cancer Institute, Rotterdam, The Netherlands; Department of Plastic and Reconstructive Surgery, University Medical Center Utrecht, Utrecht, The Netherlands; Department of Surgical Oncology and Gastrointestinal Surgery, Erasmus MC Cancer Institute, Rotterdam, The Netherlands; Department of Surgical Oncology and Gastrointestinal Surgery, Erasmus MC Cancer Institute, Rotterdam, The Netherlands; Department of Surgical Oncology and Gastrointestinal Surgery, Erasmus MC Cancer Institute, Rotterdam, The Netherlands; Department of Plastic and Reconstructive Surgery, University Medical Center Utrecht, Utrecht, The Netherlands

## Abstract

**Background:**

Malignant peripheral nerve sheath tumours (MPNSTs) have high local recurrence (LR) rates. Literature varies on LR risk factors and treatment. This study aimed to elucidate treatment options and risk factors for first and second LRs (LR1 and LR2) in a large multicentre cohort.

**Method:**

Surgically treated primary MPNSTs between 1988 and 2019 in the MONACO multicentre cohort were included. Cox regression analysed LR1 and LR2 risk factors and overall survival (OS) after LR1. Treatment of LR1 and LR2 was evaluated.

**Results:**

Among 507 patients, 28% developed LR1. Median follow-up was 66.9 months, and for survivors 111.1 months. Independent LR1 risk factors included high-grade tumours (HR 2.63; 95% c.i. 1.15 to 5.99), microscopically positive margins (HR 2.19; 95% c.i. 1.51 to 3.16) and large tumour size (HR 2.14; 95% c.i. 1.21 to 3.78). Perioperative radiotherapy (HR 0.62; 95% c.i. 0.43 to 0.89) reduced the risk. LR1 patients had poorer OS. Synchronous metastasis worsened OS (HR 1.79; 95% c.i. 1.02 to 3.14) post-LR1, while surgically treated LR was associated with better OS (HR 0.38; 95% c.i. 0.22 to 0.64) compared to non-surgical cases. Two-year survival after surgical treatment was 71% (95% c.i. 63 to 82%) *versus* 28% (95% c.i. 18 to 44%) for non-surgical LR1 patients. Most LR1 (75.4%) and LR2 (73.7%) patients received curative-intent treatment, often surgery alone (64.9% *versus* 47.4%). Radiotherapy combined with surgery was given to 11.3% of LR1 and 7.9% of LR2 patients.

**Conclusion:**

Large, high-grade MPNSTs with R1 resections are at higher LR1 risk, potentially reduced by radiotherapy. Surgically treated recurrences may provide improved survival in highly selected cases.

## Teaser

This study analyses treatment and risk factors for first and second local recurrences (LR1 and LR2) in malignant peripheral nerve sheath tumours (MPNSTs) using a large, multicentre cohort from 1988 to 2019. Findings indicate that high-grade tumours, positive margins and large tumour size increase LR1 risk, mitigated by perioperative radiotherapy. Surgically treated LR1 cases are associated with better overall survival, underscoring the need for tailored management in large, high-grade MPNSTs with R1 resections.

## Introduction

Malignant peripheral nerve sheath tumours (MPNSTs) are rare and aggressive malignant soft-tissue sarcomas (STS) and compromise 5–10% of all STS^1–3^. Approximately 50% of MPNSTs arise sporadically, while about 25–50% of MPNST cases are associated with neurofibromatosis type 1 (NF1)^4–10^. Patients with NF1 have an increased risk of developing an MPNST with a lifetime risk of 8–13%^4,11–16^. MPNSTs can originate within a (plexiform) neurofibroma in patients with NF1 and can also be present with partial rhabdomyoblastic differentiation (Triton tumour)^17^. In addition, MPNSTs can also develop sporadically or be associated with prior exposure to radiation^4,18^. Considering the various potential tumour locations, MPNSTs can exhibit a range of diverse clinical presentations. According to the European Society for Medical Oncology guidelines, the cornerstone of treatment for primary MPNST remains surgery with the aim of achieving clear surgical margins and therefore increasing survival^19^.

While there are no recommended adjuvant treatments for MPNSTs, perioperative radiotherapy (RT) is often used to improve local control^1,6,20^. On the other hand, the role of perioperative chemotherapy (CT) has not yet been fully defined. Conflicting results have been reported in the literature regarding survival benefits of CT. Despite complete resection and the use of RT, studies show that an estimated 40–70% of MPNST patients experience a first local recurrence (LR1)^7,18,20–22^. With these numbers, MPNSTs harbour among the highest recurrence rates in STS^23^. Due to its rarity, risk factors for the development of an LR1 vary in the current literature. In *Table S1*, an overview of previous larger cohort studies assessing predictors for LR has been depicted. The development of an LR1 in patients is associated with a morbid event that decreases functional outcomes^24^. As many patients have already undergone multimodal treatment (that is, surgery and RT) before experiencing a recurrence, the management of the recurrence is consequently associated with higher morbidity^25^. In certain cases, achieving local control after an LR1 may be more challenging than with primary tumours, primarily due to the distorted anatomy resulting from previous treatment^26^. There is significant value in identifying risk factors and investigating the present treatment approaches and outcomes for recurrent cases.

Overall, a diagnosis of MPNST carries a poor prognosis, and in the current literature, the treatment of recurrences remains unclear and varied^1,4,7,18,27,28^. The primary objective of treatment of recurrence is to prolong disease-free survival; nevertheless, second recurrences (LR2) do occur. Therefore, it is crucial to understand the impact of treatment options for an LR1 on the development of an LR2 and also on overall survival (OS) after an LR1.

The aim of this project was to identify risk factors associated with recurrence, and the treatment of recurrences, as well as their impact on OS in MPNST patients across nine sarcoma centres in the Netherlands and the Mayo Clinic, Rochester, Minnesota, USA; and additionally, to characterize the risk factors related to the development of an LR2 and treatment of an LR2.

## Materials and methods

### Patient population

A retrospective cohort study of the nine Dutch sarcoma centres and the Mayo Clinic, the MONACO study, was undertaken. All patients diagnosed with pathologically proven primary MPNST from 1988 to 2019 who were surgically treated for the primary tumour were included in this study. Follow-up was done according to nationwide guidelines. The diagnosis of all patients conformed to the World Health Organization’s classification of soft tissue and bone tumours^29^. Patients with uncertain pathological reports or diagnoses based on incomplete information during follow-up were excluded. Additionally, patients who presented with LR after previous resection at a different facility were excluded from the study.

### Ethical approval

The study was conducted according to the guidelines of the Declaration of Helsinki and approved by the Institutional Review Board of Erasmus Medical Center (protocol code MEC-2018-1662, 29 October 2018). Informed consent was not required due to the retrospective nature of the study and because the data were pseudo-anonymized. The study was not preregistered in an independent, institutional registry.

### Covariates

Covariates extracted from medical records for analysis were patient, tumour, and treatment characteristics and survival data. An LR1 was defined as the first radiological or pathological evidence of a recurrence at the site of the primary tumour bed. An LR2 was defined as the second radiological or pathological evidence of a recurrence at the site of the first recurrence. Age was determined as the patient's age at the time of diagnosis. The ASA classification system was employed to categorize patients’ physical status^30^. Tumour size was assessed as the maximum diameter of the tumour mass through imaging or pathology reports. Tumour grade was categorized as either low or high grade based on the Fédération Nationale des Centres de Lutte Contre le Cancer grading system, with grade 1 corresponding to a low-grade tumour, while grades 2 or 3 indicate a high-grade tumour. Tumours originating below or within the deep fascia were classified as deep-seated. NF1 status was extracted from pathological reports and was established either when explicitly mentioned in the report or when there was a pathology report of previous plexiform neurofibroma resections or the presence of two or more neurofibromas.

Surgical margin was categorized as R0 (microscopically negative, no tumour cells found in surgical borders), R1 (microscopically positive) or R2 (macroscopically positive). Tumour site was divided into extremity, central (including thorax, abdomen, pelvis, retroperitoneal) and head and neck categories. Triton status was extracted from pathological reports and was confirmed either when explicitly mentioned or when the report indicated MPNST with rhabdomyoblastic differentiation. RT-associated MPNST was defined as having previously received radiation therapy at the same site as the primary tumour bed. Concurrent metastases were defined as having metastases within 3 months after the diagnosis of a LR.

The study’s endpoints included LR1, LR2 and OS.

### Statistical analysis

All statistical analyses were performed in R (version 4.2.2). Baseline characteristics as well as treatment modalities were compared between patients with and without an LR1 and LR2.

Overall survival was defined as the duration from definitive surgery to either the date of death or the date of the last follow-up. Time-to-LR was defined as the time interval between definitive surgery and date of first LR. Time-to-LR2 was defined as the time interval between LR1 and date of LR2. Estimated median survival was calculated using the Kaplan–Meier method for several covariates of interest.

Multivariable Cox proportional hazards (PH) models were used to estimate the effect of several covariates on the development of an LR1, OS after the LR1 and the development of an LR2. In the multivariate models with LR1 or LR2 as primary outcome, death was considered as a competing risk. The selection of candidate predictors for the various outcomes was based on clinical expertise and existing literature. Univariable and multivariable analyses with 95% confidence intervals were used to estimate the effects of the covariates on the different outcomes. Variables with *P* < 0.25 from the univariable analyses were included for further evaluation when constructing the multivariable model.

Proportional hazards were assessed visually with the Schoenfeld residuals. Missing values were imputed using multiple imputations (MI; *m* = 20) and estimates were pooled using Rubin’s rule^31^. *P* ≤ 0.05 was considered statistically significant. Results from the Cox PH models were described in HRs with 95% confidence intervals. All statistical tests were two-sided. The packages ‘mice’ for MI, ‘survival’, ‘rms’ and ‘survminer’ were used for the survival and competing risk analyses.

## Results

### Patient population

**Fig. 1:**
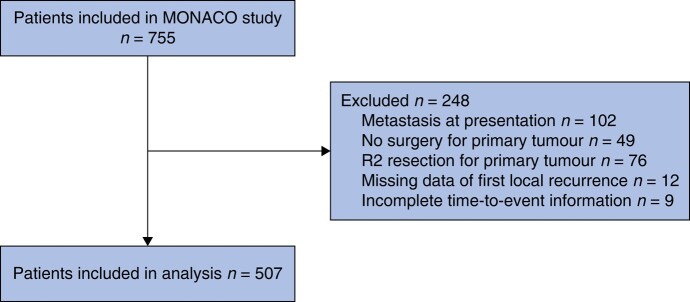
Study flow chart

A total of 755 patients were included in the MONACO database. Patients who presented with a metastasis at presentation (*n* = 102), who were not treated surgically for the primary tumour (*n* = 49), who had an R2 resection (*n* = 76), with missing data on LR1 (*n* = 12) and patients with incomplete time-to-event information (*n* = 9) were excluded in this analysis (*Fig. 1*). Of the 507 patients included in this study, 142 developed an LR1 during the follow-up period. Of the 142 patients with an LR1, patients without treatment for their recurrence (*n* = 50), patients with a metastasis during their LR1 (*n* = 13), patients with an R2 margin (*n* = 1) and patients with missing data on their LR2 (*n* = 7) were excluded from further analysis.

Patient and tumour characteristics are summarized in *Table 1*. The median follow-up time for all patients was 66.9 months (i.q.r. 108.7). The median follow-up time for survivors was 111.1 months (i.q.r. 123.1). There was a trend for a higher incidence of NF1 in patients with an LR1 (40.0% *versus* 31.1%, *P* = 0.026). In LR1 patients, there was a slight male predilection (51.4%, *P* = 0.850). Tumours were usually large (>5 cm, 53.5%, *P* < 0.005) and most were located in the extremities (50.3%, *P* = 0.140). However, it is worth noting that the male predilection and tumour location were not statistically significant. Patients who develop an LR1 often have initial high-grade tumours (92.2% *versus* 83.4%, *P* = 0.015) and microscopically positive margins (R1) (39.4% *versus* 33.2%, *P* < 0.005). Patients with an LR1 were mostly treated with surgery only for their primary tumour (44.4%) or surgery and adjuvant RT (43.0%) (*Table 2*).

**Table 1:** Patient characteristics

Variable	Overall (*n* = 507)	No LR1 (*n* = 365)	LR1 (*n* = 142)
Age (years), mean(s.d.)		43.21(20.25)	43.44(17.58)
**Male gender**	270	197 (54.1)	73 (51.4)
NA	1	1	–
**ASA**			
I	160	120 (59.1)	40 (51.3)
II	107	73 (36.0)	34 (43.6)
III	14	10 (4.9)	4 (5.1)
NA	226	162	64
**NF1**			
No	322	241 (68.9)	81 (60.0)
Yes	163	109 (31.1)	54 (40.0)
NA	22	15	7
**Tumour size**			
<5 cm	130	113 (37.5)	17 (17.0)
5–10 cm	164	117 (38.9)	47 (47.0)
>10 cm	107	71 (23.6)	36 (36.0)
NA	106	64	42
**Tumour depth**			
Superficial	73	58 (25.4)	15 (17.4)
Deep	241	170 (74.6)	71 (82.6)
NA	193	137	56
**Tumour grade**			
High grade	284	201 (83.4)	83 (92.2)
Low grade	47	40 (16.6)	7 (7.8)
NA	176	124	52
**Triton tumour**			
No	303	219 (91.2)	84 (95.5)
Yes	25	21 (8.8)	4 (4.5)
NA	179	125	54
**RT-associated**			
No	444	325 (93.4)	119 (88.1)
Yes	39	23 (6.6)	16 (11.9)
NA	24	17	7
**Site of primary tumour**			
Head and neck	71	58 (15.9)	15 (10.6)
Extremities	255	184 (50.4)	71 (50.0)
Central	177	121 (33.2)	56 (39.4)
Unknown	21	21 (5.8)	–
**Metastasis during LR1**			
No	475	365 (100.0)	110 (77.5)
Yes	32	–	32 (22.5)
**Surgical margin**			
R0	328	257 (70.4)	71 (50.0)
R1	143	87 (23.8)	56 (39.4)
Unknown	36	21 (5.8)	15 (10.6)
**Re-resection for primary tumour**			
No	365	254 (69.6)	111 (78.2)
Yes	113	81 (22.2)	22 (15.5)
NA	39	30 (8.2)	9 (6.3)

Values are *n* (%) unless otherwise indicated. LR1, first local recurrence; NA, not available; NF1, neurofibromatosis type 1; RT, radiotherapy.

**Table 2:** Initial treatment

Variable	Overall (*n* = 507)	No LR1 (*n* = 365)	LR1 (*n* = 142)
**Total treatment**			
Surgery	215	152 (41.6)	63 (44.4)
Surgery + RT	221	160 (43.8)	61 (43.0)
Surgery + CT	18	14 (3.8)	4 (2.8)
Surgery + RT + CT	53	39 (10.7)	14 (9.9)
**Any type of radiotherapy**			
No	210	151 (41.4)	59 (41.5)
Yes	280	203 (55.6)	77 (54.2)
Unknown	17	11 (3.0)	6 (4.2)
**Pre- or postoperative radiotherapy**			
No	215	154 (42.2)	61 (43.0)
nRT	74	61 (16.7)	13 (9.2)
aRT	200	138 (37.8)	62 (43.7)
Unknown	18	12 (3.3)	6 (4.2)
**Any type of chemotherapy**			
No	419	298 (81.6)	121 (85.2)
Yes	71	53 (14.5)	18 (12.7)
Unknown	17	14 (3.8)	3 (2.1)
**Pre- or postoperative chemotherapy**			
No	419	298 (81.6)	121 (85.2)
nCT	44	37 (10.1)	7 (4.9)
aCT	25	14 (3.8)	11 (7.7)
Both	2	2 (0.5)	–
Unknown	17	14 (3.8)	3 (2.1)
**Primary wound closure**			
No	41	35 (14.6)	6 (6.7)
Yes	287	204 (85.4)	83 (93.3)
**Non-functional reconstruction**			
No	386	277 (82.9)	109 (87.2)
Yes	73	57 (17.1)	16 (12.8)
NA	48	31	17
**Functional reconstruction**			
No	444	322 (95.3)	122 (97.6)
Yes	19	16 (4.7)	3 (2.4)
NA	44	27	17

Values are *n* (%). aCT, adjuvant chemotherapy; aRT, adjuvant radiotherapy; CT, chemotherapy; LR1, first local recurrence; nCT, neoadjuvant chemotherapy; nRT, neoadjuvant radiotherapy; NA, not available; RT, radiotherapy.

### Risk factors for the development of an LR1 in primary MPNST

One hundred and forty-two patients (28.0%) developed an LR1 after they underwent surgery for their primary tumour. The median time to an LR1 was 10.6 months (i.q.r. 16.7). On multivariate analysis, factors independently associated with the development of an LR1 were a high tumour grade (HR 2.63; 95% c.i. 1.15 to 5.99), microscopically positive margins (R1) (HR 2.19; 95% c.i. 1.51 to 3.16) and a tumour size >5 cm (HR 2.14; 95% c.i. 1.21 to 3.78) (*Table 3*). On the contrary, the use of RT (HR 0.62; 95% c.i. 0.43 to 0.89) reduced the risk for development of an LR1. Patients with extremity MPNSTs were more likely to receive RT (*P* = 0.004). However, in multivariate analysis, there was no significant association between the tumour location and the use of RT.

**Table 3:** Univariate and multivariate analysis of risk factors for the development of a first local recurrence

	Univariate		Multivariate	
Variables	HR (95% c.i.)	*P*	HR (95% c.i.)	*P*
Age (per 10 years)	1.01 (0.928,1.11)	0.767		
**NF1**				
No	1.00	–	1.00	–
Yes	1.49 (1.04,2.13)	0.030	1.14 (0.779,1.66)	0.507
**Tumour grade**				
Low grade	1.00	–	1.00	–
High grade	2.38 (1.10,5.16)	0.032	2.63 (1.15,5.99)	0.026
**Tumour size**				
<5 cm	1.00	–	1.00	–
>5 cm	2.45 (1.47,4.08)	0.001	2.14 (1.21,3.78)	0.011
**Triton tumour**				
No	1.00	–		
Yes	0.683 (0.271,1.73)	0.424		
**Tumour depth**				
Superficial	1.00	–	1.00	–
Deep	1.41 (0.841,2.37)	0.198	1.07 (0.607,1.90)	0.807
**Site of primary tumour**				
Head and neck	1.00	–	1.00	–
Extremities	1.26 (0.717,2.21)	0.425	1.10 (0.593,2.03)	0.768
Central	1.65 (0.934,2.93)	0.087	1.28 (0.682,2.38)	0.447
**Margin primary tumour**				
R0	1.00	–	1.00	–
R1	2.06 (1.45,2.93)	<0.001	2.19 (1.51,3.16)	<0.001
**Radiotherapy primary tumour**				
No	1.00	–	1.00	–
Yes	0.809 (0.544,1.14)	0.230	0.616 (0.426,0.892)	0.012
**Chemotherapy primary tumour**				
No	1.00	–		
Yes	0.897 (0.544,1.48)	0.669		

NF1, neurofibromatosis type 1.

### Treatment of LR1

Of the patients developing an LR1, 92 (64.9%) were surgically treated for their recurrence (*Table 4*). R0 resections were achieved in 37 (37.8%) patients. R1 resections were achieved in 13 (13.3%) patients, and three patients had an R2 margin (3.1%) as final surgical margin. LR1s were mainly treated with surgery only (50.7%). In 29 (20.4%) patients with an LR1, no treatment was performed. Among these 29 patients, the absence of treatment was likely due to the unresectability of tumours caused by tumour location (62.1% centrally located) or tumour characteristics, with most tumours being large (>10 cm, 62.5%) and high grade (94.1%). Of the 59 (41.5%) LR1 patients without primary RT, 15 (25.4%) still underwent RT for their LR1. Of the patients treated with RT, 2.8% received neoadjuvant and 14.8% adjuvant RT to surgery. In total, 5.6% of patients received only RT as treatment for their recurrence.

**Table 4:** Treatment of recurrences

Variable	LR1 (*n* = 142)	LR2 (*n* = 38)
Time to local recurrence, mean(s.d.)	23.29(34.98)	17.60(19.42)
**Surgery for LR1/LR2**		
No	44 (31.0)	14 (36.8)
Yes	92 (64.8)	23 (60.5)
Unknown	6 (4.2)	1 (2.6)
**Surgical margin**		
R0	37 (37.8)	8 (21.1)
R1	13 (13.3)	3 (7.9)
R2	3 (3.1)	1 (2.6)
Unknown	45 (45.9)	26 (68.4)
**Treatment of LR1/LR2**		
No treatment	29 (20.4)	9 (23.7)
Surgery	72 (50.7)	18 (47.4)
Surgery + RT	16 (11.3)	3 (7.9)
Surgery + CT	3 (2.1)	1 (2.6)
Surgery + RT + CT	1 (0.7)	1 (2.6)
RT	8 (5.6)	4 (10.5)
CT	7 (4.9)	1 (2.6)
Unknown	6 (4.2)	1 (2.6)
**Radiotherapy**		
No	65 (45.8)	13 (34.2)
nRT	4 (2.8)	–
aRT	21 (14.8)	8 (21.2)
Unknown	52 (36.6)	17 (44.7)
**Chemotherapy**		
No	80 (56.3)	21 (55.3)
nCT	3 (2.1)	1 (2.6)
aCT	7 (4.9)	–
Both	1 (0.7)	2 (5.3)
Unknown	51 (35.9)	14 (36.8)

Values are *n* (%) unless otherwise indicated. aCT, adjuvant chemotherapy; aRT, adjuvant radiotherapy; CT, chemotherapy; LR1, first local recurrence; LR2, second local recurrence; nCT, neoadjuvant chemotherapy; nRT, neoadjuvant radiotherapy; RT, radiotherapy.

### Risk factors for overall survival in MPNST patients with an LR1

**Fig. 2:**
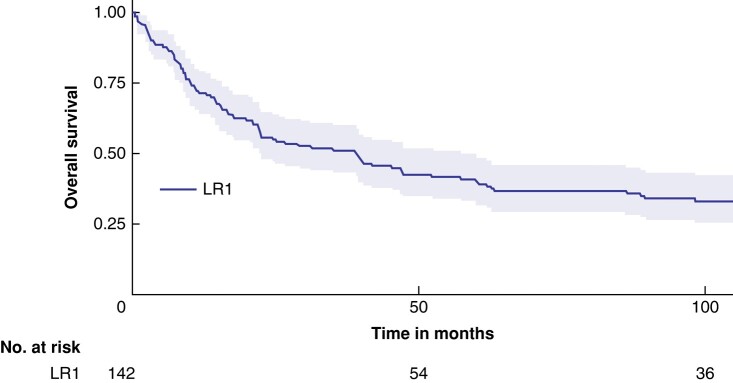
Survival after first local recurrence (LR1)

The median survival from diagnosis of an LR1 until death or last follow-up date was 39.2 months (95% c.i. 22.3 to 60.0; *Fig. 2*). Of the 142 patients with an LR1, 32 (22.5%) also had a concurrent metastasis. On multivariate analysis, factors independently associated with OS in patients with an LR1 consisted of only a metastasis during the recurrence (HR 1.79; 95% c.i. 1.02 to 3.14). Surgically treated LRs, on the other hand, were associated with better OS (HR 0.38; 95% c.i. 0.22 to 0.64; *Table 5*). The median survival in patients surgically treated for their LR was 56 months, compared to 43 months (*P* > 0.005) in patients without surgery for their LR.

**Table 5:** Univariate and multivariate analysis of risk factors for overall survival in patients with a first local recurrence

	Univariate		Multivariate	
Variables	HR (95% c.i.)	*P*	HR (95% c.i.)	*P*
Age (per 10 years)	1.05 (0.938,1.17)	0.415		
**NF1**				
No	1.00	–		
Yes	0.98 (0.614,1.53)	0.938		
**Tumour grade**				
Low grade	1.00	–	1.00	–
High grade	2.35 (1.42,3.88)	0.001	2.06 (0.846,5.00)	0.121
**Tumour size**				
<5 cm	1.00	–	1.00	–
>5 cm	1.66 (0.815,3.38)	0.170	1.24 (0.59,2.61)	0.573
**Tumour depth**				
Superficial	1.00	–	1.00	–
Deep	2.30 (1.42,3.71)	0.001	1.98 (0.985,3.96)	0.061
**Site of primary tumour**				
Head and neck	1.00	–		
Extremities	1.13 (0.570,2.25)	0.723		
Central	1.37 (0.681,2.74)	0.382		
**Margin primary tumour**				
R0	1.00	–	1.00	–
R1	1.35 (0.880,2.07)	0.174	1.08 (0.654,1.78)	0.769
**Radiotherapy primary tumour**				
No	1.00	–		
Yes	1.27 (0.830,1.94)	0.275		
**Surgery LR1**				
No	1.00	–	1.00	–
Yes	0.364 (0.238,0.557)	<0.001	0.375 (0.221,0.636)	<0.001
**Margin LR1**				
R0	1.00	–		
R1	1.39 (0.743,2.62)	0.307		
R2	0.777 (0.425,1.42)	0.418		
**Radiotherapy LR1**				
No	1.00	–	1.00	–
nRT	0.465 (0.275,0.784)	0.005	1.34 (0.526,3.40)	0.545
aRT	0.583 (0.325,1.05)	0.075	0.752 (0.388,1.46)	0.404
**Metastasis during LR1**				
No	1.00	–	1.00	–
Yes	2.532 (1.62,3.97)	<0.001	1.79 (1.02,3.14)	0.046

aRT, adjuvant radiotherapy; LR1, first local recurrence; NF1, neurofibromatosis type 1; nRT, neoadjuvant radiotherapy.

### Risk factors for the development of an LR2 and treatment

A total of 71 patients were treated with curative intent for their LR1. Among these, 38 (53.5%) patients who underwent surgical treatment for their LR1 experienced an LR2 (*Table 6*). The median time from the surgical treatment of an LR1 to the development of an LR2 was 17.6 months (i.q.r. 16.1). Of the patients who developed an LR2, 32 (84.2%) were solely treated with surgery for their LR1. A total of 12 patients also received RT following their surgery for their LR1. Among these 12 patients, 8 (33.3%) developed an LR2. Various potential risk factors for the development of an LR2 were analysed univariately (*Table 7*). However, on univariate analysis, no statistically significant risk factors contributing to the occurrence of an LR2 could be identified.

**Table 6:** Characteristics of patients with a second local recurrence

Variable	Overall (*n* = 71)	No LR2 (*n* = 33)	LR2 (*n* = 38)	*P*
Age (years), mean(s.d.)		40.15(15.32)	44.29(15.77)	0.268
Male gender	34	19 (57.6)	15 (39.5)	0.199
**ASA**				
I	27	14 (70.0)	13 (68.4)	0.576
II	11	5 (25.0)	6 (31.6)	
III	1	1 (5.0)	–	
NA	32	13	19	
**NF1**				
No	44	22 (68.8)	22 (61.1)	0.686
Yes	24	10 (31.2)	14 (38.9)	
NA	3	1	2	
**Tumour size**				
<5 cm	11	5 (21.7)	6 (25.0)	0.966
5–10 cm	10	5 (21.7)	5 (20.8)	
>10 cm	26	13 (56.5)	13 (54.2)	
NA	24	10	14	
**Tumour depth**				
Superficial	10	4 (18.2)	6 (28.6)	0.656
Deep	33	18 (81.8)	15 (71.4)	
NA	28	11	17	
**Tumour grade**				
High grade	41	22 (95.7)	19 (82.6)	0.343
Low grade	5	1 (4.3)	4 (17.4)	
NA	25	10	15	
**Triton tumour**				
Yes	44	23 (69.7)	21 (55.3)	0.982
No	1	–	1 (2.6)	
NA	26	10 (30.3)	16 (42.1)	
**Site of primary tumour**				
Head and neck	6	1 (3.0)	5 (13.2)	0.241
Extremities	44	23 (69.7)	21 (55.3)	
Central	21	9 (27.3)	12 (31.6)	
**Surgical margin LR1**				
R0	29	18 (54.5)	11 (28.9)	0.089
R1	12	4 (12.1)	8 (21.1)	
Unknown	30	11 (33.3)	19 (50.0)	
**Treatment of LR1**				
Surgery	55	23 (69.7)	32 (84.2)	0.283
Surgery + RT	12	8 (24.2)	4 (10.5)	
Surgery + CT	3	1 (3.0)	2 (5.3)	
Surgery + RT + CT	1	1 (3.0)	–	
**Radiotherapy for LR1**				
No	33	14 (42.4)	19 (50.0)	0.296
nRT	2	1 (3.0)	1 (2.6)	
aRT	11	8 (24.2)	3 (7.9)	
Unknown	25	10 (30.3)	15 (39.5)	
**Chemotherapy for LR1**				
No	40	21 (63.6)	19 (50.0)	0.642
nCT	3	1 (3.0)	2 (5.3)	
Both	1	1 (3.0)	–	
Unknown	24	10 (30.3)	14 (36.8)	

Values are *n* (%) unless otherwise indicated. CT, chemotherapy; LR2, second local recurrence; NA, not available; NF1, neurofibromatosis type 1; RT, radiotherapy.

**Table 7:** Univariate analysis of risk factors for the development of a second local recurrence

	Univariate	
Variables	HR (95% c.i.)	*P*
**Tumour grade**		
Low grade	1.00	–
High grade	0.627 (0.262,1.50)	0.308
**Tumour size**		
<5 cm	1.00	–
>5 cm	1.01 (0.376,2.69)	0.991
**Site of primary tumour**		
Head and neck	1.00	–
Extremities	0.404 (0.150,1.08)	0.081
Central	0.605 (0.213,1.72)	0.352
**Margin LR1**		
R0	1.00	–
R1	2.01 (0.832,4.87)	0.140
**Radiotherapy LR1**		
No	1.00	–
nRT	1.05 (0.376,2.92)	0.930
aRT	0.373 (0.111,1.25)	0.125
**Chemotherapy LR1**		
No	1.00	–
nCT	1.17 (0.389,3.49)	0.786
Both	1.10 (0.398,3.02)	0.861

CT, chemotherapy; LR1, first local recurrence; RT, radiotherapy.

Of the patients developing an LR2, 23 (60.5%) were surgically treated for their recurrence (*Table 4*). R0 resections were achieved in eight (21.1%) patients. R1 resections were achieved in three (7.9%) patients, and one patient had an R2 margin (2.6%) as final surgical margin. Second local recurrences were mainly treated with surgery only (47.4%). In nine patients (23.7%) with an LR2, no treatment was performed. Radiotherapy combined with surgery was administered in three (7.9%) patients and RT alone in four (10.5%). Of the patients treated with RT, all patients received adjuvant RT.

## Discussion

In patients with MPNSTs, independent risk factors for the development of an LR1 after resection are a high grade, large tumour size (>5 cm) and microscopically positive margins. The administration of radiotherapy for the primary tumour potentially reduces the risk of the development of a LR1. The treatment of LRs varied, and most patients were treated with surgery alone. Synchronous metastasis during a local recurrence had a negative impact on OS, while a surgically treated recurrence is expected to have a better OS.

According to the literature, a high tumour grade, microscopically positive margins and a large tumour size are important prognostic factors for the occurrence of an LR1 in MPNST patients, which is consistent with the findings of this study^21,32,33^. The importance of surgical quality seems crucial in the development of a recurrence^34^. While contradictory results have been reported in the literature regarding the use of RT in patients with MPNSTs, the current study demonstrates that RT reduces the risk of developing an LR1^7,11,32,35–38^. In the current literature, there is still some discussion about the use of radiotherapy in patients with MPNSTs when an R0 resection is expected^36,39,40^.

Significant factors that affect survival after an LR1 are still unknown for MPNSTs. The occurrence of concomitant metastasis during an LR1 was independently associated with worse OS following the LR1 diagnosis. Further investigation is needed to explore the relationship between histologic subtypes and LRs, as it is reasonable to assume that tumours with different subtypes may demonstrate distinct clinical behaviours and modified survival outcomes^41^.

In this cohort, a microscopically positive margin was not identified as an independent risk factor for OS after LR diagnosis. Also, tumour grade did not emerge as a significant prognostic factor influencing survival in MPNST patients after LR1. However, it should be acknowledged that the findings of this study may have been affected by a limited number of cases involving low-grade tumours (7 of 142). In contrast to other retrospective studies, tumour size was not identified as a significant factor^12,13,32,39^. The variability in the chosen cut-offs observed in other published cohorts, ranging from 5 to 15 cm, could possibly explain this discrepancy. The use of RT did not have a significant influence on survival in the current study. The current literature on the use of RT still presents inconclusive results. Some studies demonstrate improved survival in patients receiving RT, while others do not show improved long-term survival^6,12,35,39,42–45^.

A recurrence that has been treated surgically is expected to improve the 2-year survival in patients diagnosed with an LR1. This is in line with one other large cohort study (*n* = 477) in which complete surgical resection of the tumour is a significant prognostic factor for patients with recurrent STS^41^. However, it is challenging to discern whether this association between surgical treatment and the expected better OS in patients with local recurrence is due to patient selection or a genuine improvement in quality of life.

The occurrence of an LR1 after prior resection, with or without RT, significantly impacts patients’ well-being. Managing an LR1 becomes challenging due to the complexities of prior therapies and recurrence in a previously irradiated area. The treatment of recurrences depends on several factors, including the patient’s physical condition, preferences and the feasibility of curative interventions. The feasibility of a curative treatment depends on various tumour characteristics, one of which is the presence of concomitant metastasis, which is a poor prognostic factor as shown in this study. One study states that the occurrence of an LR1 is strongly influenced by the feasibility of surgical intervention for the primary tumour^46^. However, these results could be hampered by indication bias, because patients were more likely to be selected for surgery based on tumour and patient characteristics.

For primary MPNSTs, surgical resection is the recommended treatment, aiming to achieve complete removal with clear margins as the primary objective^22^. Although adjuvant or neoadjuvant therapy is being increasingly considered, its effectiveness in improving survival in primary MPNSTs has not been consistently demonstrated^47^.

Although MPNSTs generally exhibit more aggressive behaviour than most types of STS, risk factors for the development of an LR1 in other types of STS include high grade, microscopically positive margins and tumour size, consistent with findings in the current cohort^41,48^. This suggests that the same treatment strategy for recurrences may be applicable for recurrent MPNSTs as well. The authors suggest surgery as the primary treatment modality for patients with recurrent MPNSTs, while a personalized approach may be most effective for adjuvant treatment. When considering the use of RT as adjuvant treatment, it is important to take into account the disadvantages, such as wound complications in preoperative RT and late radiation toxicities in postoperative RT. These factors should be considered in the decision-making process as they can have a negative impact on functional outcome scores in patients^25,49^. Furthermore, it is important to consider that around 10% of MPNSTs can arise as a result of previous irradiation, particularly among NF1 patients^50^. This should also be taken into account during the decision-making process.

Despite curative treatment in patients with an LR1, there is still a high risk of developing an LR2. However, there is no literature available on risk factors for the development of an LR2 in MPNST patients, and only a small amount of papers have been published on LR2 in other types of STS^51–53^. Approximately 54% of patients with an LR1 requiring surgical treatment develop an LR2. This is consistent with a study investigating LR2 in patients with STS who underwent surgical treatment for their LR1, which reported a second recurrence rate of 50%^51^. Two other studies reported a LR2 rate ranging from 24% to 37% in patients with STS. In the current study, no statistically significant predictors for the development of an LR2 in patients with an LR1 were found. Most patients with an LR2 in this study underwent surgical treatment, consistent with the literature^52,53^.

This multicentre retrospective study is subject to inevitable limitations arising from its retrospective design, including potential selection bias due to selective loss of follow-up and missing data. However, over 90% of the included patients were followed until death, and a multiple imputation technique was used to reduce this risk of bias. Due to its retrospective nature, patients in this study underwent treatment over a span of nearly 30 years, potentially leading to variations in treatment standards that could impact the results. Additionally, it is important to acknowledge that a central review of pathology was not performed in this study, which could introduce limitations in accurately diagnosing MPNST due to the absence of specific histologic criteria. Also, due to the low number of patients treated for an LR1 and subsequently developing an LR2, it is likely that univariate analyses could not find any significant risk factors.

Nevertheless, due to the size of this large international and nationwide study on recurrent MPNST, new insights have been provided. Furthermore, as all included patients were treated in specialized centres, the review of pathology might be of lesser significance. This design enhances the generalizability of the data and models by minimizing the potential for selection or referral bias. As STS can present very heterogeneously, research on a single histological subtype level is necessary to improve our understanding of tumour behaviour to aid tailoring ideal treatment and outcomes. In contrast to most population-based studies on (recurrent) MPNST, this study incorporated significant entity-specific details, including NF1 and Triton status, as well as important clinical and treatment information on LRs.

Almost 30% of the MPNST patients develop an LR. Consistent with the literature, this study demonstrated that risk factors associated with a higher risk of a recurrence were a high grade, microscopically positive margin and larger tumour size. The use of RT was associated with a reduced risk of development of a recurrence. The treatment of LRs varied, and most patients were treated with surgery only. Synchronous metastasis during an LR1 had a negative impact on survival, while surgically treated cases showed longer OS. Despite curative treatment of an LR1, 54% will develop an LR2.

## Collaborators

MONACO collaborators: J. Henk Coert, Uta E. Flucke, Willem-Bart M. Slooff, Thijs van Dalen, Lukas B. Been, Han J. Bonenkamp, Monique H. M. E. Anten, Martinus P. G. Broen, Marc H. A. Bemelmans, Jos A. M. Bramer, Gerard R. Schaap, Arthur J. Kievit, Winan J. van Houdt, Jos van der Hage and Michiel A. J. van de Sande.

## Supplementary Material

zrae024_Supplementary_Data

## Data Availability

The data that support the findings of this study are available on request from the corresponding author, upon reasonable request. The data are not publicly available due to information that could compromise the privacy of research participants.
