# Supplementary material for: Local recurrence in malignant peripheral nerve sheath tumours: multicentre cohort study
Source: BJS Open. 2024 Apr 15;8(2):zrae024. doi: 10.1093/bjsopen/zrae024 (PMC11018273; doi:10.1093/bjsopen/zrae024)
Supplement: zrae024_Supplementary_Data [file zrae024_supplementary_data.docx]

**Local recurrence in malignant peripheral nerve sheath tumours: multicentre cohort study**

*Christianne Y.M.N. Jansma^1,2*^, Ibtissam Acem^1^, Dirk J. Grünhagen^1^, Cornelis Verhoef^1^, Enrico Martin^2^, and MONACO Collaborators^†^*

*^1^ Department of Surgical Oncology and Gastrointestinal Surgery, Erasmus MC Cancer Institute, Dr. Molewaterplein 40, 3015 GD Rotterdam, The Netherlands;* [*c.jansma@erasmusmc.nl*](mailto:c.jansma@erasmusmc.nl) *;* [*i.acem@erasmusmc.nl*](mailto:i.acem@erasmusmc.nl) *;* [*d.grunhagen@erasmusmc.nl*](about:blank) *;* [*c.verhoef@erasmusmc.nl*](about:blank) *;*

*^2^ Department of Plastic and Reconstructive Surgery, University Medical Center Utrecht, Heidelberglaan 100, 3584 CX Utrecht, The Netherlands;* [*E.Martin-2@umcutrecht.nl*](about:blank)

*^*^ Correspondence:* [*c.jansma@erasmusmc.nl*](mailto:c.jansma@erasmusmc.nl)

*^†^ MONACO Collaborators is provided in the Acknowledgments.*

**Corresponding Author Name & Email Address**

Christianne Jansma, PhD-student

Department of Surgical Oncology and Gastrointestinal Surgery

Erasmus Medical Center Cancer Institute

Dr Molewaterplein 40

3015 GD Rotterdam

The Netherlands

Phone: +31-017040704

Email: [c.jansma@erasmusmc.nl](mailto:c.jansma@erasmusmc.nl)

ORCID: <https://orcid.org/0009-0000-5881-9316>

**Supplementary Materials - Index**

| **Supplementary Figures and Tables** |  |
| --- | --- |
| Table 1. Overview of predictors for the development of an LR1 in cohort studies | *pag. 2* |
| **References** |  |
|  | *pag, 3* |

**Supplementary Figures and Tables**

| **Supplementary Table 1**. Overview of predictors for the development of an LR1 in cohort studies | | | | | | | | | | |
| --- | --- | --- | --- | --- | --- | --- | --- | --- | --- | --- |
| **Factors influencing Risk of LR1** | | | | | | | | | | |
| **Study** | **n of patients** | **Type of STS** | **Analysis** | **NF1** | **Grade** | **Tumor size** | **Depth** | **Site** | **Margin (R1)** | **RT** |
| Current study | 499 | MPNST | MV | NS | + | + | NS | NS | + | + |
| Stucky et al.^1^ | 175 | MPNST | UV | NS | NS | NS | NS | NS | NS | NS |
| Anghileri et al.^2^ | 205 | MPNST | MV | NS | + | + | NA | + | + | NS |
| Wang et al.^3^ | 43 | MPNST | MV | NA | + | NS | NA | NA | NS | NS |

LR1: first local recurrence, MV: multivariate analysis, NF1: neurofibromatosis type 1, NA: not available, NS: not significant, n: number of patients, RT: radiotherapy, +: significant, UV: univariate analysis

**References**

1. Stucky, C.-C.H., et al., *Malignant peripheral nerve sheath tumors (MPNST): the Mayo Clinic experience.* Annals of surgical oncology, 2012. **19**: p. 878-885.
2. Anghileri, M., et al., *Malignant peripheral nerve sheath tumors: prognostic factors and survival in a series of patients treated at a single institution.* Cancer: Interdisciplinary International Journal of the American Cancer Society, 2006. **107**(5): p. 1065-1074.
3. Wang, T., et al., *Malignant peripheral nerve sheath tumor (MPNST) in the spine: a retrospective analysis of clinical and molecular prognostic factors.* Journal of neuro-oncology, 2015. **122**: p. 349-355.
